# Investigation of Sociodemographic Characteristics and Psychosocial Risk Factors in Adolescents Diagnosed with Major Depressive Disorder and Their Effects on Suicide Probability

**DOI:** 10.1192/j.eurpsy.2025.445

**Published:** 2025-08-26

**Authors:** M. Dilli Gürkan, S. G. Sismanlar

**Affiliations:** 1 Child and Adolescent Psychiatry, Bandırma Education and Research Hospital, Balıkesir; 2 Child and Adolescent Psychiatry, Kocaeli University, Kocaeli, Türkiye

## Abstract

**Introduction:**

Those with a history of depression during adolescence are at increased risk of suicidal ideation, attempts, and suicide completion in adulthood. Therefore, in order to reach adolescents at risk of suicide attempt or death due to suicide, it is important to determine the risk factors for MDD and predictors of suicidal ideation in adolescents diagnosed with major depressive disorder (MDD) in clinical samples.

**Objectives:**

This study aimed to compare adolescents diagnosed with major depressive disorder with healthy adolescents in terms of sociodemographic characteristics and psychosocial risk factors and to determine the important predictors of suicidal ideation based on sociodemographic variables.

**Methods:**

The study group included 53 adolescents aged between 15 and 18 years who were newly diagnosed with major depressive disorder (MDD) 53 adolescents matched in terms of age and gender, who did not have a mental disorder diagnosis.Schedule for Affective Disorders and Schizophrenia for School-Age Children (6-18 Years) - Present and Lifetime Version - DSM-5 was applied to the adolescents by the clinician, and the Sociodemographic Data Form was filled in with the information obtained from both adolescents and parents. Adolescents were asked to fill out the Suicide Probability Scale.

**Results:**

In our study, adolescents diagnosed with major depressive disorder (MDD) were found to have higher rates of smoking, alcohol use, broken family structure, and school absenteeism compared to the control group; while the family’s monthly income, the child’s academic success, the rate of attending daycare during childhood, and their participation in social activities were found to be lower. Alcohol use and family relationship were found to be factors that increase the likelihood of suicide in adolescents diagnosed with depression. In the study, adolescents diagnosed with depression were found to have a higher rate of physical abuse, self-harm behavior, and a history of suicide attempts, domestic physical violence, and a history of suicide attempts among friends compared to the control group. Domestic physical violence was found to be factors that increase the likelihood of suicide in adolescents diagnosed with depression.

**Conclusions:**

In our study, we tried to define the sociodemographic differences and possible risk factors of depression in adolescents diagnosed with depression who applied to a university hospital clinic compared to healthy adolescents.Conducting large-sample community-based studies to understand the risk factors for depression and suicidal thoughts, which are common in children and adolescents, is very important in terms of community mental health and preventive mental health.

**Disclosure of Interest:**

M. Dilli Gürkan: None Declared, S. Sismanlar Consultant of: editing the text

